# Biochemical and transcriptome analyses of a novel chlorophyll-deficient chlorina tea plant cultivar

**DOI:** 10.1186/s12870-014-0352-x

**Published:** 2014-12-10

**Authors:** Lu Wang, Chuan Yue, Hongli Cao, Yanhua Zhou, Jianming Zeng, Yajun Yang, Xinchao Wang

**Affiliations:** Tea Research Institute, Chinese Academy of Agricultural Sciences, Hangzhou, 310008 China; National Center for Tea Plant Improvement, Hangzhou, 310008 China; Key Laboratory of Tea Biology and Resources Utilization, Ministry of Agriculture, Hangzhou, 310008 China

**Keywords:** Chlorina, Chlorophyll deficiency, Gene expression, Microarray, Tea plant (*Camellia sinensis*)

## Abstract

**Background:**

The tea plant (*Camellia sinensis* (L.) O. Kuntze) is one of the most economically important woody crops. Recently, many leaf color genotypes have been developed during tea plant breeding and have become valuable materials in the processing of green tea. Although the physiological characteristics of some leaf color mutants of tea plants have been partially revealed, little is known about the molecular mechanisms leading to the chlorina phenotype in tea plants.

**Results:**

The yellow-leaf tea cultivar Zhonghuang 2 (ZH2) was selected during tea plant breeding. In comparison with Longjing 43 (LJ43), a widely planted green tea cultivar, ZH2 exhibited the chlorina phenotype and displayed significantly decreased chlorophyll contents. Transmission electron microscopy analysis revealed that the ultrastructure of the chloroplasts was disrupted, and the grana were poorly stacked in ZH2. Moreover, the contents of theanine and free amino acids were significantly higher, whereas the contents of carotenoids, catechins and anthocyanin were lower in ZH2 than in LJ43. Microarray analysis showed that the expression of 259 genes related to amino acid metabolism, photosynthesis and pigment metabolism was significantly altered in ZH2 shoots compared with those of LJ43 plants. Pathway analysis of 4,902 differentially expressed genes identified 24 pathways as being significantly regulated, including ‘cysteine and methionine metabolism’, ‘glycine, serine and threonine metabolism’, ‘flavonoid biosynthesis’, ‘porphyrin and chlorophyll metabolism’ and ‘carotenoid biosynthesis’. Furthermore, a number of differentially expressed genes could be mapped to the ‘theanine biosynthesis’, ‘chlorophyll biosynthesis’ and ‘flavonoid biosynthesis’ pathways. Changes in the expression of genes involved in these pathways might be responsible for the different phenotype of ZH2.

**Conclusion:**

A novel chlorophyll-deficient chlorina tea plant cultivar was identified. Biochemical characteristics were analyzed and gene expression profiling was performed using a custom oligonucleotide-based microarray. This study provides further insights into the molecular mechanisms underlying the phenotype of the chlorina cultivar of *Camellia sinensis*.

**Electronic supplementary material:**

The online version of this article (doi:10.1186/s12870-014-0352-x) contains supplementary material, which is available to authorized users.

## Background

Tea plant (*Camellia sinensis* (L.) O. Kuntze), which is cultivated for the production of a non-alcoholic beverage, is one of the most economically important woody crops worldwide [1]. There are great genetic variations in tea plant germplasms [2,3]. Many leaf color cultivars have recently been developed during tea plant breeding and have become valuable materials in the processing of green tea. For example, ‘Baiye 1 (Anji Baicha)’ and ‘Xiaoxueya’, two albino tea cultivars grown in China, exhibit white young shoots when the environmental temperature is below 20°C in the early spring [4,5]. The development of chloroplasts and the accumulation of chlorophyll *a* and *b* are blocked in these two albino cultivars at the albinistic stage [5-7]. In addition to the leaf color changes corresponding to lower chlorophyll contents in ‘Baiye 1’ and ‘Xiaoxueya’, there are also changes in the leaf chemical composition, which is an important determinant of the sensory quality and healthy effects of tea [4,8]. Higher total free amino acid concentrations are detected in the leaves of these two cultivars compared with regular green tea leaves [4,8]. The albino tea cultivars have received increased attention and are popular with tea consumers in China because of their unique leaf color and abundant free amino acids.

Among chlorophylls, carotenoids and flavonoids/anthocyanins, which are the three main pigment classes in leaf tissue, chlorophyll and carotenoids are the principal pigments that trap light energy in photosynthetic organisms. It has been demonstrated that the development of chlorotic leaves is related to chlorophyll metabolism and chloroplast development [5,7,9-12]. The biosynthesis of chlorophyll occurs in the grana in chloroplasts. A series of enzymatic steps are involved in chlorophyll biosynthesis, which involve the conversion of glutamate to chlorophyll *a* and chlorophyll *b* [13]. The chlorophyll biosynthetic pathway is divided into three main parts: (i) the formation of 5-aminolevulinic acid (ALA), (ii) the formation of protoporphyrin IX from eight molecules of ALA, and (iii) the Mg-protoporphyrin pathway producing chlorophyll [14]. Large numbers of leaf color mutants with chlorophyll deficiencies have been identified in plants, and many efforts have been made to elucidate the regulatory pathways affecting chlorophyll biosynthesis.

It has been reported that a barley mutant lacking chlorophyll *b* shows a chlorina phenotype due to a lack of enzymes converting chlorophyll *a* into chlorophyll *b* [15]. The inbred maize line A661 exhibits a dramatic reduction in its chlorophyll content when grown at a temperature below 15°C [16]. The chelation of Mg^2+^ in protoporphyrin IX to form the Mg-protoporphyrin complex is an important step that is unique to chlorophyll production. The enzyme catalyzing this insertion is known as Mg-chelatase, which is composed of three subunits, ChlD, ChlH and ChlI, and when any one of the subunits is mutated, Mg-chelatase activity is abolished [14,17,18]. Chlorina mutants defective in genes encoding the Mg-chelatase subunits have been identified in various plants [14]. The mutants *Arabidopsis ch42-3* and *gun5* lack *ChlI* and *ChlH,* respectively, and are deficient in chelating the Mg^2+^ into protoporphyrin IX [19,20]. Rice plants with mutations in *OsChlH*, *OsChlD* and *OsChlI* display the chlorina phenotype [9,11]. Barley mutants lacking *ChlH*, *ChlD* or *ChlI* are unable to synthesize chlorophyll [10]. Either a reduction or excess accumulation of *ChlI* in tobacco results in the loss of chlorophyll [21]. In addition to genes encoding Mg-chelatase, other genes related to chlorotic leaves have also been identified, such as *OsYGL1*, which encodes a chlorophyll synthase responsible for catalyzing the esterification of chlorophyllide, *OsYLC1*, which is a chloroplast-localized gene, and *OsLYL1*, which encodes a geranylgeranyl reductase [12,22,23]. These genes influence the biosynthesis of chlorophyll and alter chlorophyll contents.

Plants with reduced levels of chlorophyll are the ideal genetic material for exploring the molecular mechanisms regulating the chlorina phenotype and chloroplast development. Compared with studies in model plants with known genome sequences, the lack of genomic information for tea plants has cause molecular biology research in this species to lag behind. Although the physiological characteristics of some leaf color genotypes of tea plants have been partially revealed, little is known about the molecular mechanisms regulating chlorophyll deficiencies in tea plants.

In this study, we compared the biochemical characteristics of two tea cultivars, the normal green tea cultivar Longjing 43 (LJ43) and the novel chlorophyll-deficient chlorina cultivar Zhonghuang 2 (ZH2). The chlorophyll contents of the leaves and the ultrastructure of the chloroplasts of the two cultivars were studied. Furthermore, the levels of carotenoids, theanine, amino acids and flavonoids in the leaves were measured. To reveal the underlying molecular mechanism related to the differences between the two tea cultivars, we used a custom oligonucleotide-based microarray [24] to investigate gene expression in the two cultivars, and identified differentially expressed genes, including genes related to amino acid metabolism, photosynthesis, and pigment metabolism.

## Methods

### Plant material and sample preparation

Two tea plant (*Camellia sinensis* (L.) O. Kuntze) cultivars, ‘Longjing 43 (LJ43)’ and ‘Zhonghuang 2 (ZH2)’, were used in this study. The plants were 5 years old and had been grown in the field at the Tea Research Institute of the Chinese Academy of Agricultural Sciences (TRI, CAAS, N 30°10′, E 120°5′), Hangzhou, China. LJ43 is a normal green tea cultivar that is widely planted in China, especially in Zhejiang province. ZH2 is a yellow-leaf cultivar that was selected from a natural yellow-leaf mutant in Zhejiang province via systematic selection.

For chemical assays and microarray analysis, the firstly new sprouting fresh shoots (two leaves and one bud) were sampled in spring, and three independent biological replicates were performed. Each replicate was collected from more than ten randomly selected tea plants. The samples were divided into two duplicate, one was steamed for 5 min, dried at 80°C and ground into powder for chemical assay, and the other one was stored at −80°C after flash-freezing with liquid nitrogen for microarray analysis.

### Measurement of chlorophyll, beta-carotene and lutein contents

Chlorophyll was extracted with 80% acetone from 100 mg dried samples. The extract was measured spectrophotometrically at 645 nm and 663 nm. The chlorophyll content was determined according to the method of Arnon [25], while the lutein and beta-carotene contents were measured via HPLC as described previously [26].

### Measurement of theanine, free amino acids, catechins and anthocyanin

To measure theanine and catechins, 100 mg of the dried samples was extracted at 90°C for 30 min in 50 ml of water, with shaking once every 10 min. The filtrates were diluted, then refiltered through a 0.45 μm nylon filter and analyzed via HPLC [27]. The anthocyanin content was measured as described previously [28].

For the measurement of total free amino acids, the ninhydrin colorimetric method was used. Briefly, a 3 g dried sample was extracted in a boiling water bath for 45 min in 450 ml of H_2_O (with shaking once every 10 min). After filtration, the volume of the filtrates was increased to 500 ml by adding H_2_O, and 1 ml of the solution was transferred to a 25 ml flask. Following the addition of 0.5 ml of buffer (pH 8.0) containing 63 mM Na_2_HPO_4_ and 3 mM KH_2_PO_4_, 0.5 ml of a 2% ninhydrin solution (2 g ninhydrin and 80 mg SnCl_2_.2H_2_O dissolved in 100 ml of water) was added, and the flask was incubated in a boiling water bath for 15 min. The volume was then increased to 25 ml with H_2_O. The absorption values of the solution at 570 nm were determined using a spectrophotometer. The total free amino acid content was calculated from a standard curve generated with varying concentrations of glutamine.

### Transmission electron microscopic (TEM) analysis

The ultrastructure of the chloroplasts was investigated via TEM according to the method described by Du et al. [5]. Leaf samples (fresh shoots) were cut into small pieces and fixed with 2.5% glutaraldehyde overnight at 4°C. The samples were then washed with 0.1 M phosphate buffer (pH 7.0) three times (15 min each) and subsequently refixed in 1% (v/v) OsO_4_ for 2 h and washed with 0.1 M phosphate buffer again. For dehydration, the fixed samples were subjected to a graded ethanol series (50%, 70%, 80%, 90% and 95%), with each dehydration step lasting 15 min, and then soaked in 100% ethanol for 20 min. The dehydrated samples were drenched in acetone for 20 min, followed by epoxy resin and acetone (v/v = 3/1) for 1 h. The samples were finally imbedded in pure epoxy resin at 70°C overnight.

After imbedding, 70–90 nm thick sections were cut with a Reichert-Jung ultra-cut microtome (Reichert-Jung, Heidelberg, Germany). The sections were stained with saturated uranyl acetate in 50% ethanol and 0.2% (w/v) lead citrate for 15 min each. The images were examined under a JEM-1230 microscope (JEOL, Akishima, Tokyo, Japan).

### Microarray data analysis

Total RNA was extracted from 0.5 g to 1 g of leaf samples from ZH2 and LJ43 tea plants as described previously [29] for microarray and qRT-PCR assays. The 60-mer oligonucleotide probes and microarray were designed by eArray (Agilent). The probes on the microarray were based on the 42,440 unigene sequences obtained from a 454 RNA-seq dataset in our laboratory [24]. The microarray data were deposited into the NCBI Gene Expression Omnibus (GEO) database under accession number GSE52255 [24].

Feature Extraction Software (10.7.1.1, Agilent Technologies) was used to analyze array images to obtain raw data. GeneSpring was employed to complete the basic analysis of the raw data. The raw data were normalized with a quantile algorithm. The probes with at least one condition out of every condition flagged in “*P*” were chosen for further data analysis. Differentially expressed genes were subsequently identified based on the observed fold changes and using a *t*-test-calculated *P*-value. A differentially expressed gene was defined as a variation in the gene expression test showing a *P*-value < 0.05 and a fold change > 2. Significant pathways were identified based on the Kyoto Encyclopedia of Genes and Genomes (KEGG). Fisher’s exact test was employed to select pathways, and the threshold of significance was defined as a *P*-value < 0.05 and an false discovery rate (FDR) < 0.05.

### Quantitative Real-Time RT-PCR

Five micrograms of RNA was used to synthesize cDNA. The RNA samples were treated with RNase-free DNase I (amplification grade, Invitrogen, Carlsbad, CA, USA) to remove residual genomic DNA. First-strand cDNA was synthesized using SuperScript® III Reverse Transcriptase (Invitrogen, Carlsbad, CA, USA). Real-time quantitative RT-PCR was performed with the Applied Biosystems 7500 Sequence Detection System (Carlsbad, CA, USA) using SYBR® Premix Ex Taq™ II (TaKaRa Biomedicals). The amplification efficiency of the primers for the tested genes is shown in Additional file 1. Triplicate quantitative assays were performed on each cDNA sample, and the 18S rRNA reference gene was used as an internal control. Relative transcript levels were calculated relative to the level of 18S rRNA using the 2^-ΔΔCt^ formula [30]. All data are presented as the mean ± SD (*n* = 3). All of the primer sequences used for qRT-PCR are shown in Additional file 2.

### Statistical analysis of the data

The data were expressed as the means ± SD from three independent biological replicates. Significance was determined via one-way analysis of variance, and for differences between groups, the least significant difference (LSD) *t*-test was employed (*P* < 0.05).

## Results

### Growth performance of a chlorophyll-deficient chlorina cultivar

**Figure 1:**
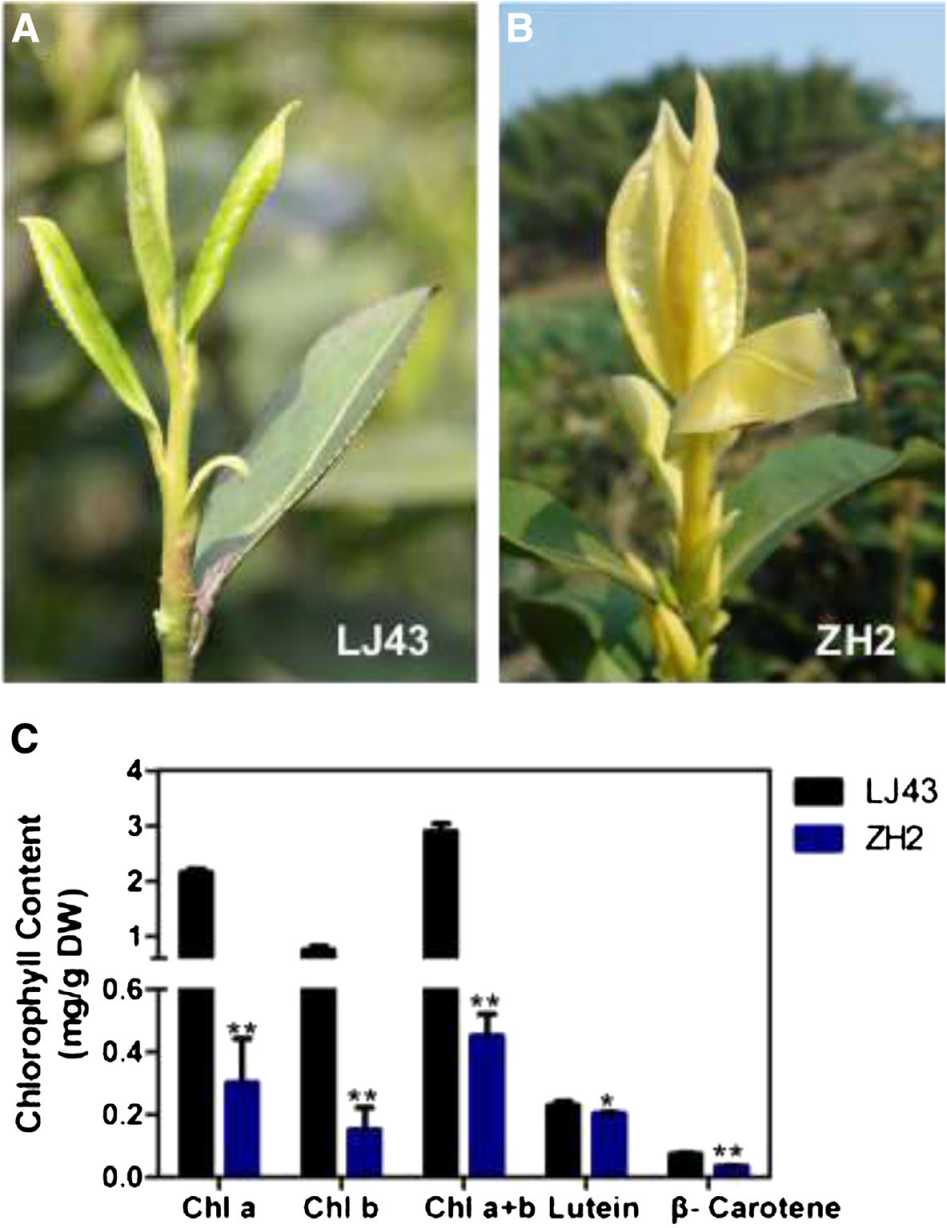
**Growth performance and pigment (chlorophyll and carotenoid) contents of LJ43 and ZH2. (A)** Growth performance of LJ43 shoots in spring. **(B)** Growth performance of ZH2 shoots in spring. **(C)** Chlorophyll *a* (Chl a), chlorophyll *b* (Chl b), Chl a + b, lutein and β-carotene contents of two leaves and one bud of LJ43 and ZH2. The significance of differences compared with LJ43 is indicated with an asterisk (*P* < 0.05) or two asterisks (*P* < 0.01).

A yellow-leaf cultivar was developed from a natural mutant and was designated ZH2. In spring, ZH2 exhibits yellow new shoots, which differs from what occurs in LJ43, a green tea cultivar that is widely planted in China (Figure 1A and B). It is well known that leaf greening is the result of chlorophyll biosynthesis, and the chlorophyll contents of two leaves and one bud of the tea plants were measured. The results showed that in ZH2, both chlorophyll *a* and chlorophyll *b* contents were significantly lower than in LJ43 (approximately 14% and 20% of the contents in LJ43, respectively; Figure 1C). These results suggested that the yellow leaves of ZH2 result from reduced chlorophyll levels and that the lower chlorophyll content of ZH2 might result from abnormal chlorophyll biosynthesis.

Carotenoids are lipid-soluble pigments that are essential components of the photosynthetic apparatus. Beta-carotene and lutein, the major carotenoids found in tea leaves, are two nutritionally important plant-derived carotenoids [31]. The rice pigment-deficient mutant *ylc1* exhibits a chlorosis phenotype with decreased levels of chlorophyll and lutein [22]. Beta-carotene and lutein contents were measured in the two cultivars. The results showed that in the young shoots of ZH2, the beta-carotene and lutein contents were decreased to 48.8% and 88.6% of the levels in LJ43, respectively (Figure 1C).

### Ultrastructure of chloroplasts in LJ43 and ZH2

**Figure 2:**
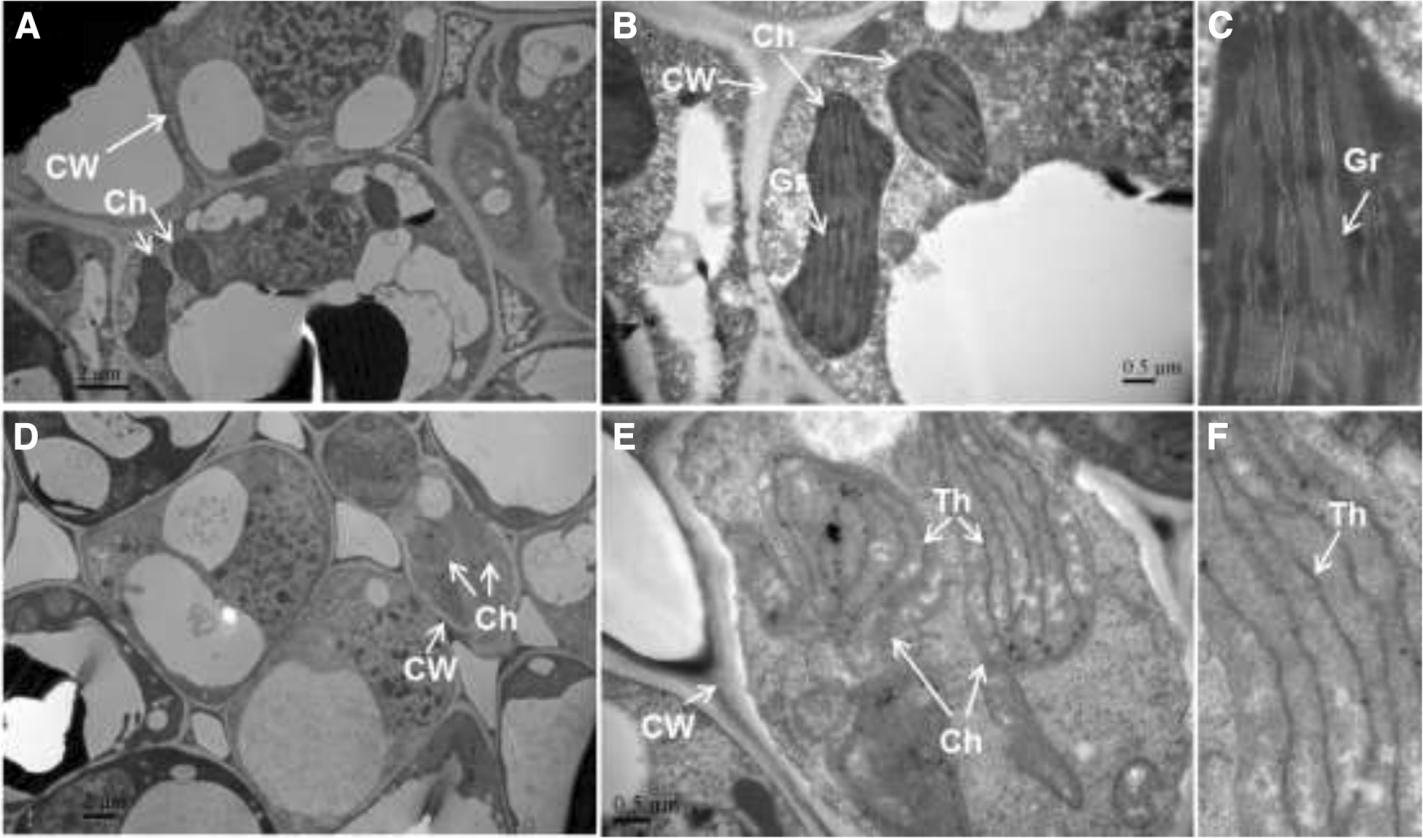
**Comparison of subcellular structures in the young leaves of LJ43 and ZH2. (A, B, C)** Subcellular structures of LJ43. **(D, E, F)** Subcellular structures of ZH2. **(C, F)** Magnification of the chloroplasts from **B** and **E**, respectively. Ch: Chloroplast, CW: Cell Wall, Gr: Grana, Th: Thylakoid.

Photosystems involve chlorophyll and other pigments, which are located in the grana. The saclike membranes that make up grana are known as thylakoids. To understand why yellow leaves occur in ZH2, the ultrastructure of the chloroplast, which is responsible for leaf greening, was investigated through TEM. TEM analysis showed a typical ultrastructure, consisting of grana and thylakoids, in the chloroplasts of LJ43 plants (Figure 2A to C). However, in the chloroplasts of ZH2, the stacks of grana disappeared, and only a few thylakoids remained (Figure 2D to F). This result indicated that the change in the leaf color of ZH2 might be a consequence of damage to the development of grana.

### Theanine, amino acid and flavonoid contents of LJ43 and ZH2

**Figure 3:**
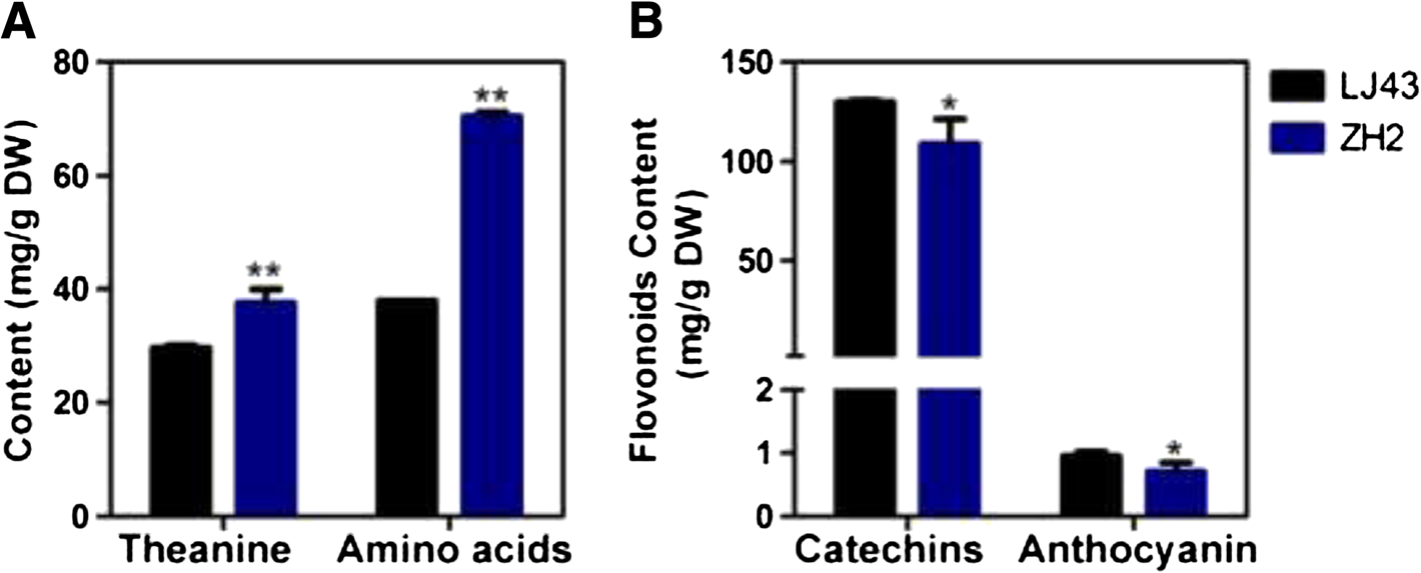
**Theanine, amino acid, catechins and anthocyanin contents of LJ43 and ZH2. (A)** Theanine and amino acid contents of two leaves and one bud of the two cultivars. **(B)** Catechins and anthocyanin contents in two leaves and one bud of the two cultivars. The significance of differences compared with LJ43 is indicated with an asterisk (*P* < 0.05) or two asterisks (*P* < 0.01).

Amino acids are important in the sensory quality of green tea [32]. Theanine, which was first discovered in tea leaves, is the most abundant free amino acid in tea plants and is beneficial for human health [33-37]. The theanine and total free amino acids contents in the shoots (two leaves and one bud) of LJ43 and ZH2 were analyzed. The results showed that the contents of both theanine and total free amino acids were significantly higher in the leaves of ZH2 than in LJ43 (Figure 3A).

Flavonoids are important for tea quality and are synthesized through a branched pathway that yields both colorless compounds (e.g., flavonols) and colored pigments (e.g., anthocyanins) [38]. Catechins represent 60% to 80% of the total flavonoids in green tea [39]. Measurement of the contents of catechins and anthocyanins in shoots revealed that they were lower in ZH2 than in LJ43 (Figure 3B).

### Microarray analysis of gene expression

**Figure 4:**
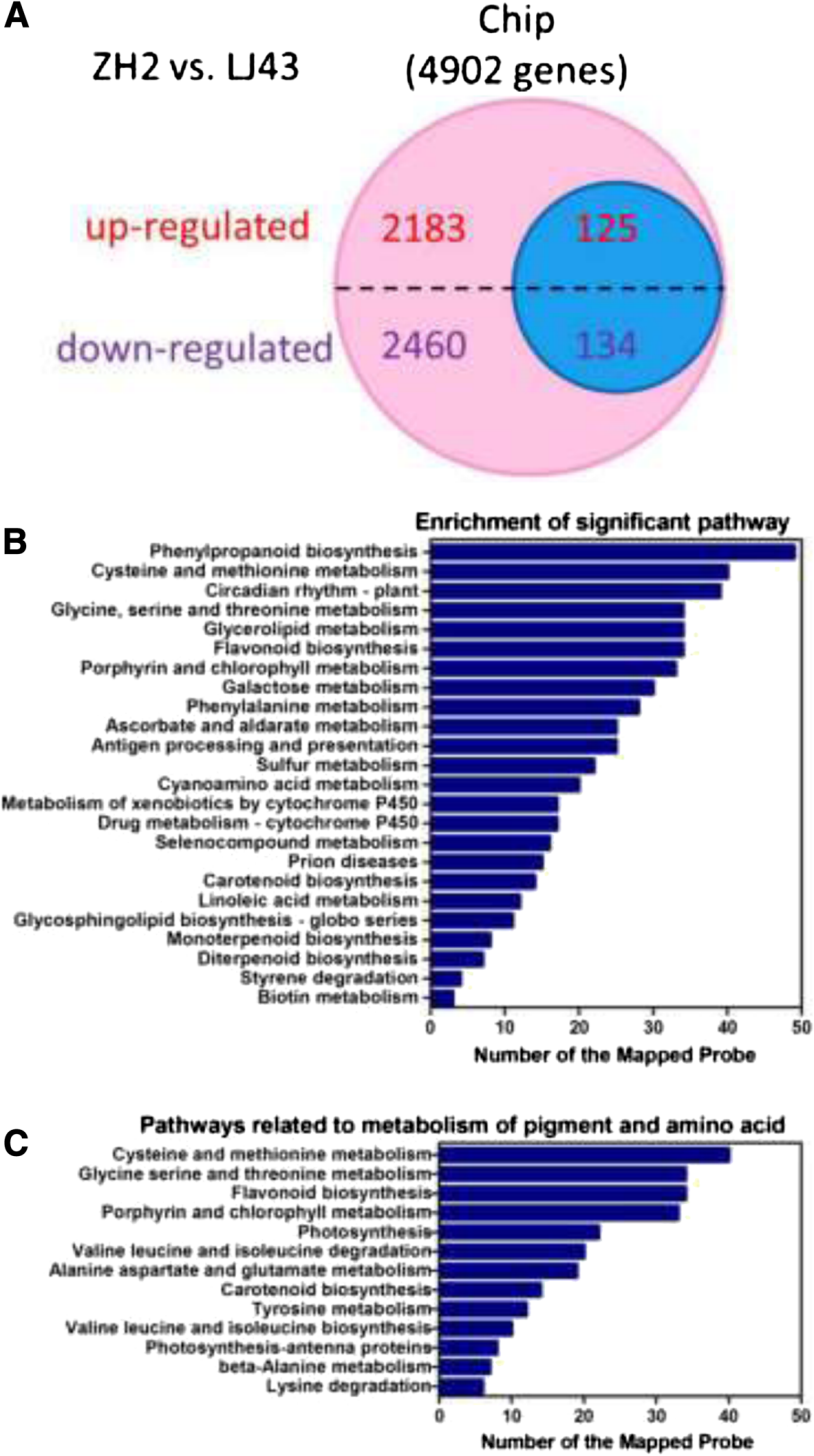
**Differential expression analyses. (A)** Diagram showing the number of significantly differentially expressed genes (*P*-value < 0.05, fold change > 2) in ZH2 compared with LJ43 (ZH2 versus LJ43 - pink and blue circles). Differentially expressed genes related to photosynthesis and amino acid and pigment metabolism (ZH2 versus LJ43 – blue circle). **(B)** Significantly (*P*-value < 0.05, FDR < 0.05) enriched pathways (based on KEGG) among the 4,902 differentially expressed genes. **(C)** KEGG-based pathway assignments of the 259 differentially expressed genes (ZH2 versus LJ43 – blue circle in **A)** related to photosynthesis and amino acid and pigment metabolism.

To investigate the performance of ZH2, the differences in gene expression between ZH2 and LJ43 were studied to elucidate the reason for the different phenotype of ZH2 plants. A custom microarray was used to assess global gene expression in ZH2 and LJ43. The number of genes that were significantly (*P* < 0.05) differentially expressed in ZH2 by more than 2 fold compared with LJ43 are listed in Figure 4A. Altogether, 4,902 differentially expressed genes were identified, including 2,308 genes that were up-regulated and 2,594 that were down-regulated in ZH2 (Figure 4A). Genes related to amino acid metabolism, pigment (chlorophyll, carotenoid and flavonoid) metabolism and photosynthesis are indicated in the blue circle (125 up-regulated and 134 down-regulated) (Figure 4A, Additional file 3).

An evaluation of functional category enrichment based on gene ontology (GO) was performed on the genes that were differentially expressed (*P*-value < 0.05, fold-change > 2) between ZH2 and LJ43. Significant GO terms were determined at a cut-off *P*-value < 0.05. AgriGO annotated 3,182 out of 4,902 differentially expressed genes with at least one significant GO term, and the annotated probes were classified according to the ‘biological process’, ‘molecular function’ and ‘cellular component’ categories and their sub-categories (Table 1). Among the three components, the ‘metabolic process’ sub-category of the ‘biological process’ category accounted for the majority of GO annotations, followed by ‘binding’ under ‘molecular function’ (Table 1).

**Table 1:** **Categorization of differentially expressed genes between ZH2 and LJ43**

**GO terms**	**Differentially expressed genes**			**Ratio (%)**
	**Total**	**Up-regulated**	**Down-regulated**	
**Cellular component**				
GO: 0016020 membrane	924	436	488	29.04
GO: 0005623 cell	485	226	259	15.24
GO: 0005576 extracellular region	130	41	89	4.09
GO: 0030054 cell junction	1	1	0	0.03
**Biological process**				
GO: 0008152 metabolic process	1758	820	938	55.25
GO: 0051179 localization	493	232	261	15.49
GO: 0050896 response to stimulus	289	113	176	9.08
GO: 0009987 cellular process	331	146	185	10.4
GO: 00650007 biological regulation	196	98	98	6.16
GO:0071840 cellular component organization or biogenesis	33	9	24	1.04
GO:0032502 developmental process	20	9	11	0.63
GO:0051704 multi-organism process	5	3	2	0.16
GO:0032501 multicellular organismal process	4	0	4	0.13
**Molecular function**				
GO: 0005488 binding	1010	473	537	31.74
GO: 0005215 transporter activity	116	62	54	3.65
GO: 0009055 electron carrier activity	112	44	68	3.52
GO: 0016209 antioxidant activity	26	16	10	0.82
GO: 0045735 nutrient reservoir activity	18	11	7	0.57
GO: 0005198 structural molecule activity	1	0	1	0.03

Note that an individual probe might be assigned to more than one GO term.

To identify biological pathways, the genes were annotated with the corresponding enzyme commission (EC) numbers from BLASTX alignments against the KEGG database. By associating the 4,902 differentially expressed genes with Gene IDs in KEGG, 255 pathways were mapped (Additional file 4). Among these pathways, 24 pathways, including ‘cysteine and methionine metabolism’, ‘glycine, serine and threonine metabolism’, ‘flavonoid biosynthesis’, ‘porphyrin and chlorophyll metabolism’ and ‘carotenoid biosynthesis,’ were considered significant at a cut-off *P*-value < 0.05 and FDR < 0.05 (Figure 4B). These pathways are suggested to be important to the unusual phenotype of ZH2. The 259 genes (Additional file 3) from the up- and down-regulated datasets presented in Figure 4A (blue circle) were classified into 12 pathways, and the enrichment of each pathway is shown in Figure 4C.

**Figure 5:**
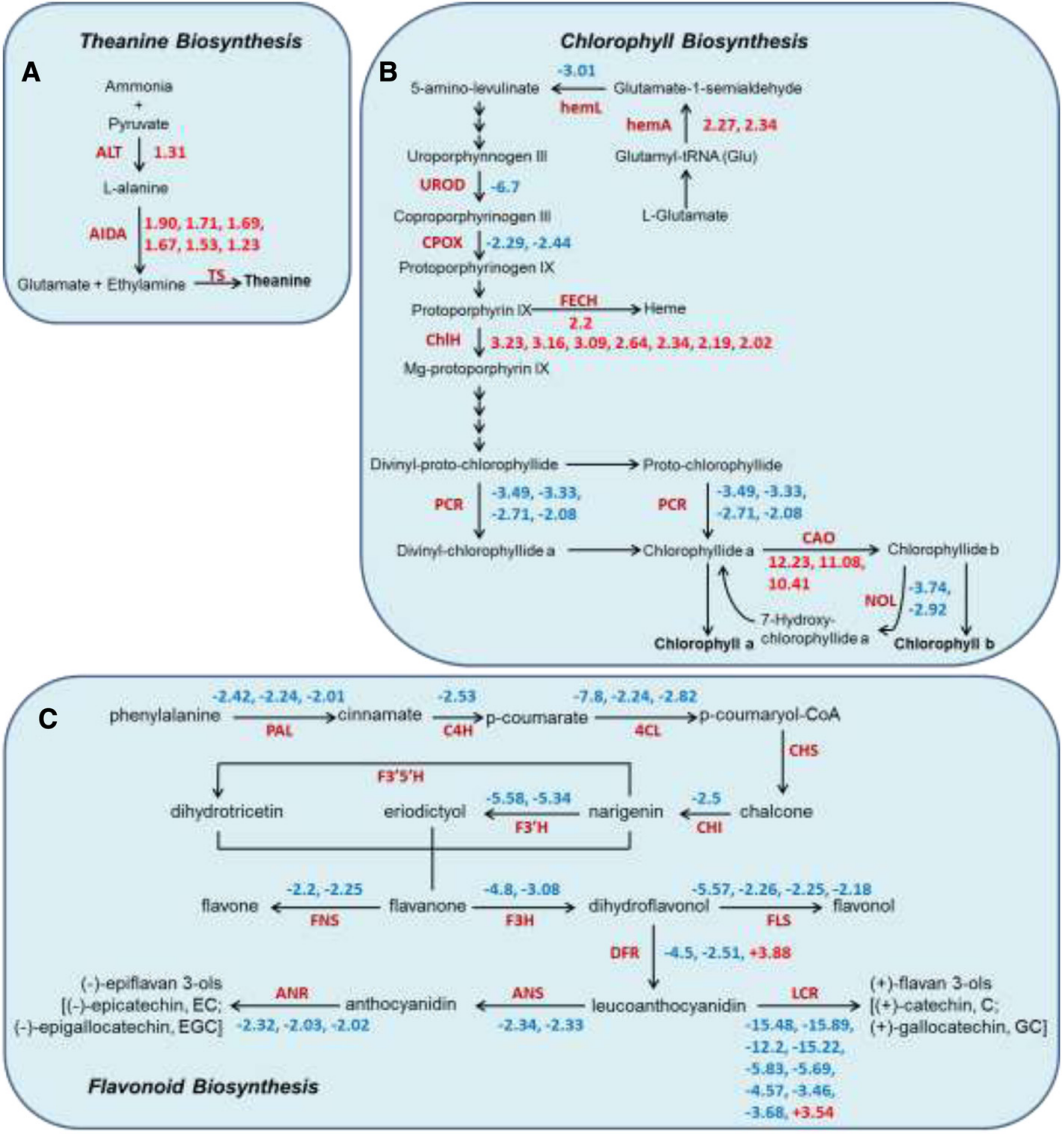
**Schematic representation of significant changes in the transcript levels of genes involved in three pathways. (A)** Genes involved in the ‘theanine biosynthesis’ pathway (note that AIDA was selected from the homologues of arginine decarboxylase, ADC). **(B)** Genes involved in the ‘chlorophyll biosynthesis’ pathway. **(C)** Genes involved in the ‘flavonoid biosynthesis’ pathway. The ‘flavonoid biosynthesis’ pathway was drawn according to Shi *et al.* [41]. The numbers represent the fold change; positive numbers indicate significantly (*P* < 0.05) up-regulated genes, while negative numbers indicate significantly (*P* < 0.05) down-regulated genes.

When pathways of interest were examined, several differentially expressed genes could be mapped to the ‘theanine biosynthesis’, ‘chlorophyll biosynthesis’ and ‘flavonoid biosynthesis’ pathways. Theanine is synthesized from glutamic acid and ethylamine by theanine synthetase (*TS*) [40]. Ethylamine appears to be produced from alanine by alanine decarboxylase (*AIDA*) [40]. *TS* and *AIDA* were not found in our dataset. The *AIDA* shown in Figure 5A was selected from the homologues of arginine decarboxylase *(ADC*), which exhibits similar domains to *AIDA* [41,42]. Alanine transaminase (*ALT*) and *AIDA* were significantly (*P*-value < 0.05) up-regulated in ZH2, indicating the possibility that the higher contents of theanine found in ZH2 resulted from higher transcription levels of the two genes involved in theanine biosynthesis (Figure 5A).

Nine genes in the chlorophyll biosynthesis pathway showed differential expression (*P*-value < 0.05, fold change > 2; 4 up-regulated and 5 down-regulated genes). The expression of some critical successive enzymes for converting 5-amino-levulinate (ALA) to chlorophyll *a* was altered to variable extents in ZH2 (Figure 5B). ChlH, which is a subunit of Mg-chelatase, was up-regulated in ZH2. Transcripts encoding enzymes such as those functioning in early enzymatic steps, from the formation of glutamate-1-semialdehyde to protoporphyrin IX, showed lower levels. Critical enzymes for converting Mg-protoporphyrin IX to chlorophyll were also inhibited (Figure 5B).

Compared with LJ43, ZH2 exhibited lower contents of catechins and anthocyanin (Figure 3B). To examine whether the expression of genes related to ‘flavonoid biosynthesis’ was altered in ZH2, we searched the differentially expressed genes using EC numbers and found that genes involved in ‘flavonoid biosynthesis’ were significantly down-regulated in ZH2 (Figure 5C), which is consistent with the lower catechins and anthocyanin contents observed in the young shoots of ZH2.

### Quantitative RT-PCR validation of some differentially expressed genes

**Figure 6:**
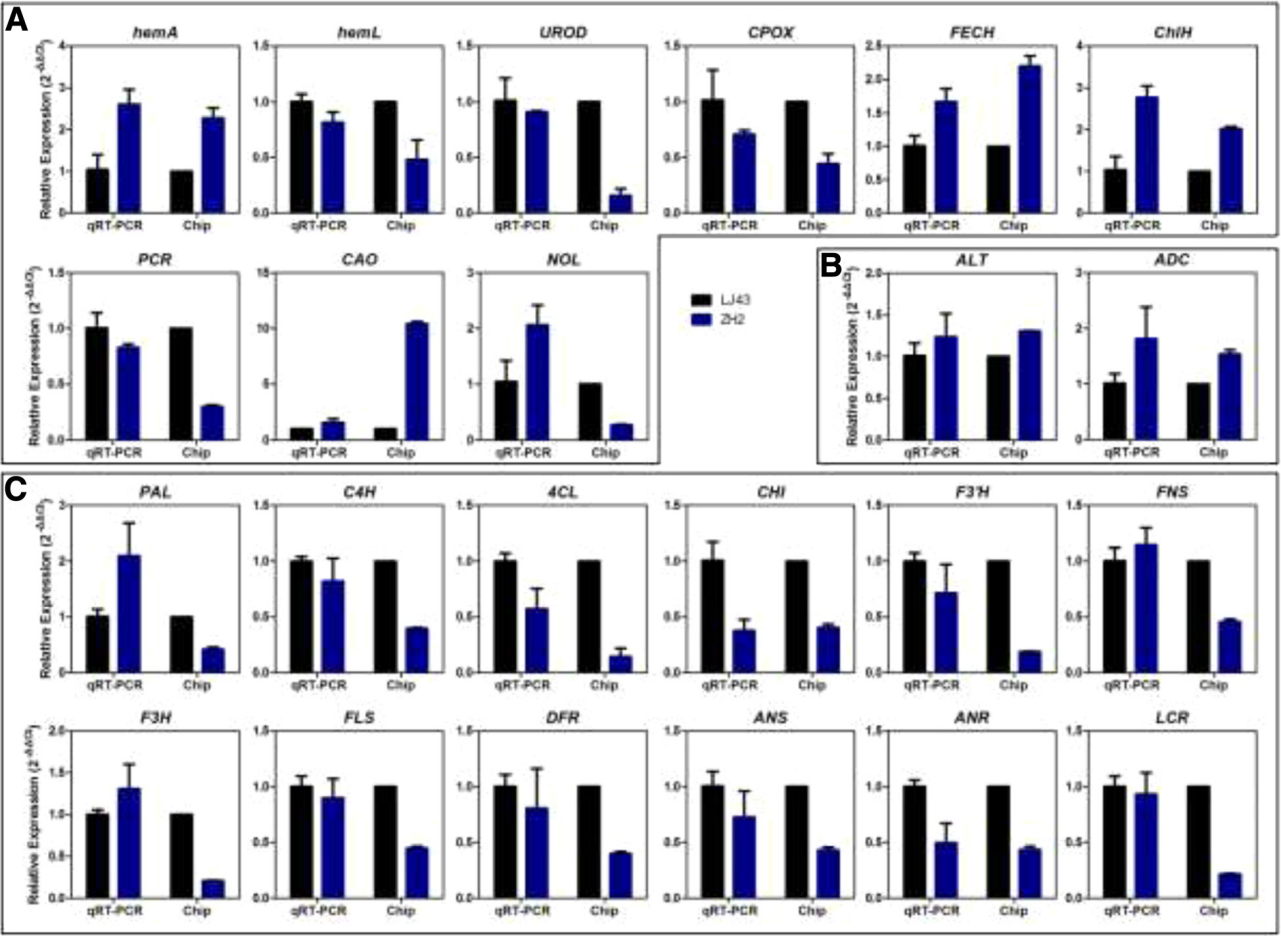
**Gene expression patterns in the shoots of LJ43 and ZH2. (A)** Genes involved in the ‘chlorophyll biosynthesis’ pathway. **(B)** Genes involved in the ‘theanine biosynthesis’ pathway. **(C)** Genes involved in the ‘flavonoid biosynthesis’ pathway. All data are shown as the mean ± SD (*n* = 3).

To confirm the microarray data and the expression changes in genes related to ‘chlorophyll biosynthesis’, ‘theanine biosynthesis’ and ‘flavonoid biosynthesis’, qRT-PCR was performed. The expression of 23 genes (including 2 genes involved in ‘theanine biosynthesis’, 9 genes involved in ‘chlorophyll biosynthesis’ and 12 genes involved in ‘flavonoid biosynthesis’) was detected. The expression of 19 genes detected via qRT-PCR showed a pattern that was similar to that observed in the microarray data, whereas 4 genes (*CsNOL, CsPAL, CsFNS, CsF3H*) showed a different expression pattern (Figure 6). Ten genes randomly selected from these 23 genes were cloned by RACE to support that the differentically expressed genes detected by qRT-PCR in the two cultivars were believable (Additional file 5). Overall, the qRT-PCR results were well correlated with the microarray data.

## Discussion

Tea cultivars exhibiting unique color changes have drawn increasing attention due to the differences in the chemical composition of their leaves. In this study, we report a novel chlorophyll-deficient chlorina tea cultivar that presents a yellow new shoot phenotype (Figure 1). The ZH2 cultivar was selected during tea plant breeding, and genetic and biochemical characterization of ZH2 was performed.

Pigment analysis showed that the young shoot phenotype of ZH2 is positively correlated with the levels of chlorophyll, beta-carotene and lutein (Figure 1). It seemed that the arrested development of grana in the chloroplasts blocks chlorophyll and carotenoids biosynthesis in ZH2 and the photosynthesis was inhibited in ZH2 leaves. Recently, Feng et al. found that decreased abundances of carotenoids and chlorophylls were accompanied by an increase in the abundances of free amino acids in albino tea leaves [43]. This finding is in accordance with our results, which showed that the contents of chlorophyll and the carotenoids of beta-carotene and lutein were decreased in ZH2 (Figure 1C). Chlorophylls and carotenoids are important for the biogenesis of the photosynthetic apparatus. Carotenoids are synthesized in the membranes of nearly all types of plastids, including chloroplasts [44,45]. In chloroplasts, carotenoids form photosynthetic complexes in thylakoid membranes, and they play an important role in protecting the chlorophyll from destruction [46,47]. Thus, it is reasonable that reduction in levels of carotenoids were accompanied with the reduction in levels of chlorophylls and the abnormal pigments contents were along with the aberrant development of chloroplast. Also, the result will be helpful for furthering our understanding the chlorina phenomenon in relation to carotenoid biosynthesis and the development of the photosynthetic apparatus.

Changes in chlorophyll contents may be one reason for yellow leaves. Changes in the expression of genes involved in ‘chlorophyll biosynthesis’ might result in the chlorina phenotype of ZH2. Mg-chelatase plays an important role in chlorophyll production. In plants, this enzyme complex consists of three subunits, designated ChlD, ChlH and ChlI. Mg-chelatase deficiency is a common denominator in many chlorophyll-deficient mutants. Plants lacking *ChlH* exhibit defective chlorophyll and show a chlorina phenotype [9,10,20]. Although ZH2 presents a leaf phenotype that is similar to mutants such as *Oschlh*, the expression of *CsChlH* was found to be up-regulated in ZH2 (Figures 5B and 6A). Considering the other two subunits of Mg-chelatase, ChlD and ChlI, which, when mutated, can also cause a chlorina phenotype in plants [11,19], we examined the expression of *CsChlD* and *CsChlI* in the microarray data. We found that *CsChlI* (ZH2 versus LJ43, *P*-value = 0.011, fold change = 1.16) and *CsChlD* (ZH2 versus LJ43, *P*-value = 0.000031, fold change = 1.27) were also slightly up-regulated in ZH2. This result indicated that the expression of the three subunits of Mg-chelatase was not disrupted in ZH2 and that the chlorina phenotype of ZH2 was not correlated with a lack of Mg-chelatase subunits, as has been reported in other plants. However, it is likely that there were changes in these genes at the posttranscriptional level that might affect the ZH2 phenotype.

In *Camellia sinensis*, Ma (2012) analyzed differentially expressed genes in different albescent stages of the ‘Anji Baicha’ cultivar using a 5 K cDNA microarray and found that *CsChlH* was up-regulated at the pale white shoot stage, consistent with our findings [48]. It has been reported that both decreased and increased expression of *ChlI* decreases Mg-chelatase activity and reduces chlorophyll synthesis in transgenic tobacco plants [21]. Active Mg-chelatase requires a balanced proportion of each subunit, and an excess of the ChlI subunit could disturb the correct assembly of the enzyme complex [21]. In the present study, *CsChlH* showed a higher level of induction than *CsChlD* and *CsChlI* in ZH2, which might upset the balance of the three subunits and consequently influence Mg-chelatase activity in ZH2.

Genes encoding enzymes involved in the early enzymatic steps of the ‘chlorophyll biosynthesis’ pathway were found to be suppressed in ZH2 (Figure 5B). Lower activities related to ALA synthesis and the resulting lower protoporphyrin IX contents are directly correlated with reduced Mg-chelatase activity [49]. *CAO* is required for chlorophyll *b* synthesis, and the expression of *CAO* is up-regulated in plants that do not have sufficient chlorophyll *b* [50]. The present study revealed an increased transcript level of the *CsCAO* gene in ZH2, which is consistent with its lower chlorophyll *b* content (Figures 1C and 5B). Interestingly, the chlorina phenotype of ZH2 disappeared under shade treatment (unpublished data) and we speculate that the chlorina phenotype with chlorophyll metabolic disorder in ZH2 may be regulated by light. Shade treatment could be performed to investigate the effect of low light on the chlorina phenotype and gene expression pattern in the future.

Based on microarray analysis, differentially expressed genes were identified. Twenty-four pathways were identified as significantly regulated, many of which were related to amino acid metabolism and pigment metabolism, which was strongly correlated with the different chlorophyll and amino acid contents observed in ZH2 (Figure 4B). Furthermore, many secondary metabolic processes, such as ‘flavonoid biosynthesis’, that are important for plant growth and development were significantly enriched [51]. It has been reported that the catechins content decreases during the albescent process in tea plants, which is consistent with our findings [43,52]. Genes involved in ‘flavonoid biosynthesis’ were repressed in ZH2 (Figure 5C). This repression might be responsible for the lower amounts of the anthocyanin pigments and catechins found in ZH2 (Figure 3B). Anthocyanin/flavonoids are responsible for the red, blue and purple anthocyanin pigments of plant tissues [53]. It is possible that the decreased levels of anthocyanin pigments might be related to the changes in leaf color observed in ZH2. Several secondary metabolic processes, though not ‘flavonoid biosynthesis’, were also significantly enriched (Figure 4B), which suggested that the secondary metabolites of ZH2 were altered, and it is therefore of interest to study the metabolic profile of ZH2 in the future.

In this study, we compared biochemical components and the transcriptomes of one chlorina cultivar (ZH2) and one normal green tea plant cultivar (LJ43) and found that there were many differences in biochemical components as well as in transcriptome profiles. Microarray analysis revealed that some differentially expressed genes could be mapped to the ‘theanine biosynthesis’, ‘chlorophyll biosynthesis’ and ‘flavonoid biosynthesis’ pathways based on KEGG. Changes in the expression of genes involved in these three pathways might be responsible for the higher theanine content and the chlorina phenotype of ZH2. Thus, this study provides further insights into the molecular mechanisms underlying the phenotype of this chlorina cultivar of *Camellia sinensis*. However, it is possible that the genes annotated in this study might not function as expected because the genome sequence information for the tea plant is not available, and we cannot rule out the possibility that our analysis of differentially expressed genes might be influenced by inappropriate annotation. As ZH2 and LJ43 do not share the same genetic background, many of the changes that we identified in this study might be presumptive and preliminary. Nevertheless because phenotypic traits such as the contents of free amino acids, catechins, flavonoids and pigments are controlled by genetic factors, the differences between ZH2 and LJ43 might result from changes in gene expression. We also found that the chlorina phenotype was heritable in the F_1_ offspring of ZH2 plants, and the chlorina phenomenon observed in ZH2 is influenced by light intensity and time. Therefore, the results obtained in this study may provide new insights toward elucidating the essential mechanism underlying the chlorina phenotype of tea plants. In the future, we will carry out further research to elucidate the regulatory factors (e.g., environmental and genetic factors) that control and regulate the chlorina phenotype of ZH2.

## Conclusions

In summary, the physiological characteristics of the novel chlorophyll-deficient chlorina cultivar Zhonghuang 2 (ZH2) were analyzed, and gene expression profiling was performed using a 4X 44 K custom oligonucleotide-based microarray (GSE52255). The lower chlorophyll contents and abnormal ultrastructure of chloroplasts observed in the leaves of ZH2 suggested that chlorophyll biosynthesis was partially inhibited. As observed in other leaf color mutant cultivars of *Camellia sinensis*, ZH2 exhibited higher contents of theanine and free amino acids. Differential expression analysis revealed 4,902 genes that were differentially expressed in the two cultivars, and 24 pathways were identified as significantly differentially regulated, including amino acid metabolism and pigment metabolism pathways, which might be related to the higher amino acid content and chlorophyll-deficient phenotype of ZH2. Further analysis revealed that a number of differentially expressed genes could be mapped to the ‘theanine biosynthesis’, ‘chlorophyll biosynthesis’ and ‘flavonoid biosynthesis’ pathways based on KEGG. Changes in the expression of genes involved in these three pathways might be responsible for the higher theanine content and the chlorina phenotype of ZH2. Our study provides further insights into the molecular mechanisms underlying the phenotype of this chlorina cultivar of *Camellia sinensis*.

## Additional files

Additional file 1:
**Amplification efficiency of the genes tested by qRT-PCR.**
Additional file 2:
**Primer sequences for quantitative RT-PCR.**
Additional file 3:
**The 259 differentially expressed genes related to amino acid metabolism, pigment (chlorophyll, carotenoid and flavonoid) metabolism and photosynthesis.**
Additional file 4:
**The 255 pathways that mapped to the differentially expressed genes between ZH2 and LJ43.**
Additional file 5:
**Ten genes randomly selected from microarray data were cloned from two tea cultivars by RACE.**

## Abbreviations

ADC: Arginine decarboxylase
AIDA: Alanine decarboxylase
ALA: Ainolevulinic acid
ALT: Alanine transaminase
ANR: Anthocyanidin reductase
ANS: Anthocyanidin synthase
CAO: Chlorophyllide a oxygenase
C4H: Cinnamate 4-hydroxylase
CHI: Chalcone isomerase
ChlH: Magnesium chelatase subunit H
CHS: Chalcone synthase
4CL: 4-coumarate CoA ligase
CPOX: Coproporphyrinogen oxidase
DFR: Dihydroflavonol 4-reductase
EC: Enzyme commission
FDR: False discovery rate
FECH: Ferrochelatase
F3H: Flavanone 3-hydroxylase
F3′H: Flavonoid 3′-hydroxylase
F3′5′H: Flavonoid 3′,5′-hydroxylase
FLS: Flavonol synthase
FNS: Flavone synthase
GEO: Gene expression omnibus
GO: Gene ontology
hemA: Glutamyl-tRNA reductase
hemL: Glutamate-1-semialdehyde aminotransferase
KEGG: Kyoto encyclopedia of genes and genomes
LCR: Leucoanthocyanidin reductase
NOL: Chlorophyll b reductase
PAL: Phenylalanine ammonia lyse
PCR: Protochlorophyllide reductase
TEM: Transmission electron microscopic
TS: Theanine synthetase
UROD: Uroporphyrinogen III decarboxylase
