# Supplementary material for: Biochemical and transcriptome analyses of a novel chlorophyll-deficient chlorina tea plant cultivar
Source: BMC Plant Biol. 2014 Dec 10;14:352. doi: 10.1186/s12870-014-0352-x (PMC4276261; doi:10.1186/s12870-014-0352-x)
Supplement: Additional file 1: — Amplification efficiency of the genes tested by qRT-PCR. [file 12870_2014_352_MOESM1_ESM.docx]

**Additional file**

**Additional file 1 Amplification efficiency of the primers for genes tested by qRT-PCR**

| **Gene** | **Standard Curve** | **R^2^** | **E value** |
| --- | --- | --- | --- |
| ***ALT*** | Y=-3.4435X+29.356 | 0.9992 | 0.9516 |
| ***ADC*** | Y=-3.4915X+30.336 | 0.9955 | 0.9338 |
| ***hemA*** | Y=-3.5925X+29.101 | 0.9995 | 0.8983 |
| ***hemL*** | Y=-3.521X+27.259 | 0.9959 | 0.9231 |
| ***UROD*** | Y=-3.438X+27.763 | 0.9994 | 0.9537 |
| ***CPOX*** | Y=-3.655X+32.277 | 1 | 0.8776 |
| ***FECH*** | Y=-3.505X+29.724 | 0.9930 | 0.9289 |
| ***ChlH*** | Y=-3.634X+29.191 | 0.9990 | 0.8844 |
| ***PCR*** | Y=-3.034X+29.193 | 0.9941 | 1.1360 |
| ***CAO*** | Y=-3.442X+31.234 | 0.9940 | 0.9522 |
| ***NOL*** | Y=-3.7065X+27.668 | 1 | 0.8612 |
| ***PAL*** | Y=-3.7315X+31.784 | 0.9931 | 0.8535 |
| ***C4H*** | Y=-3.5505X+27.492 | 0.9996 | 0.9127 |
| ***4CL*** | Y=-3.713X+28.82 | 0.9997 | 0.8592 |
| ***CHI*** | Y=-3.621X+29.233 | 1 | 0.8887 |
| ***F3’H*** | Y=-3.655X+30.821 | 0.9990 | 0.8776 |
| ***FNS*** | Y=-3.6815X+31.848 | 0.9993 | 0.8691 |
| ***F3H*** | Y=-3.4635X+26.304 | 0.9988 | 0.9441 |
| ***FLS*** | Y=-3.4035X+25.347 | 0.9986 | 0.9528 |
| **DFR** | Y=-3.5695X+28.982 | 0.9996 | 0.9061 |
| **ANR** | Y=-3.72X+28.308 | 0.9999 | 0.8570 |
| **ANS** | Y=-3.5135X+31.422 | 1 | 0.9258 |
| **LCR** | Y=-3.0125X+22.247 | 0.9914 | 1.1476 |
| **18S rRNA** | Y=-3.4265X+23.218 | 0.9907 | 0.9581 |
