# Supplementary material for: Biochemical and transcriptome analyses of a novel chlorophyll-deficient chlorina tea plant cultivar
Source: BMC Plant Biol. 2014 Dec 10;14:352. doi: 10.1186/s12870-014-0352-x (PMC4276261; doi:10.1186/s12870-014-0352-x)
Supplement: Additional file 2: — Primer sequences for quantitative RT-PCR. [file 12870_2014_352_MOESM2_ESM.docx]

**Additional file**

**Additional file 2 Primer sequences for quantitative RT-PCR**

| **Genes** | **Forward Primer (5’ - 3’)** | **Reverse Primer (5’- 3’)** |
| --- | --- | --- |
| **Cs18S** | TCTCAACCATAAACGATGCCGACCAG | TTTCAGCCTTGCGACCATACTCCC |
| **hemA** | CATTTCAACAGGTGTAGTGTGG | CGCTCGAACAACTCAAGGCATC |
| **hemL** | GGTTATATAAGCGGGATGTTTG | CAGTGTCACTCTTCTTCGCATC |
| **UROD** | GAGGAGTCAGTTCCGTATGTTG | AACAAACCCAGTACGGCCGCTT |
| **CPOX** | TTGGACTCAAGACGGGAGGTAG | GTTTCCATTCTTCGCTTCCCTC |
| **FECH** | AGTTCTTCAACGCTTCCAAGAG | CCACTTTCATTTCAGATTTGGC |
| **chlH** | GGGATGTTTGGGTGAATAGTCC | TTCCTTCCCAAGAAAACCAACC |
| **PCR** | TGACTTGCTCTCTTTGGGGATG | ATCCATGGCTCTCCAGTCTGCT |
| **CAO** | ACGTGTCTGGTCCTGTTCAACC | GTGTCATCCTTCAAGTCCGTAG |
| **NOL** | TTTCACGGATGCCATGGAGATG | ATTATGAAGGCACAAACGACAG |
| **ALT** | GGTGCACAATACTGCCTCAAGA | CAATGAATCCCTTGTGGAAGTC |
| **ADC** | AGAAGCTCGGGTTTTGACCCT | CCTGTGAAATGCAACCAAGAC |
| **PAL** | ACAGGAGAGAGAATTAGATCAC | GTGCACCATTCCAATCCTTGAG |
| **C4H** | GAGCATGGACAACAATAGTCTG | ACGTTGTCTTCATTGATCTCTC |
| **4CL** | TACAAAGGGTTCCAAGTAGCTC | AACTTCTCCTGCAGCCTCATCT |
| **CHI** | CAGAATTGTTGAAGGAGAAACC | TCAGCAACCTAACCACTAGTGT |
| **F3’H** | GCAATGATTTCGAGCTCATACC | CTAGTGTGCCCAAAATGTACTC |
| **FNS** | AGGACGAGATAAAACTATCAGG | CATAGTGAAGAACTTGCAGTCC |
| **F3H** | CTGGAAACGATGAATCCACCTT | GACTCGCTCGTGAGTTTTTTGC |
| **FLS** | GTCATGCACCCTCCTAAGAAG | CTGTCACTCCCCTGTATGAAG |
| **DFR** | TCCCATGATGCTACCATCCATG | TCAGTGGGGACATTGTACTCAG |
| **ANS** | GGCCTTGAATCGATTCCAAAAG | TTCTCTTCCTCGAATACGTTGC |
| **ANR** | CCATACCCTCCAGTGCTACGAG | TTGTGAAGATGTTGCTAGAGAG |
| **LCR** | CAGAATCATGTTGTTGAAGTAC | AGATGGATTAGATATGGTTCAC |
