# Supplementary material for: Biochemical and transcriptome analyses of a novel chlorophyll-deficient chlorina tea plant cultivar
Source: BMC Plant Biol. 2014 Dec 10;14:352. doi: 10.1186/s12870-014-0352-x (PMC4276261; doi:10.1186/s12870-014-0352-x)

**Additional file 5**

**Ten genes randomly selected from microarray data were cloned from two tea cultivars by RACE.** Primers for qRT-PCR were indicated by red box.

**
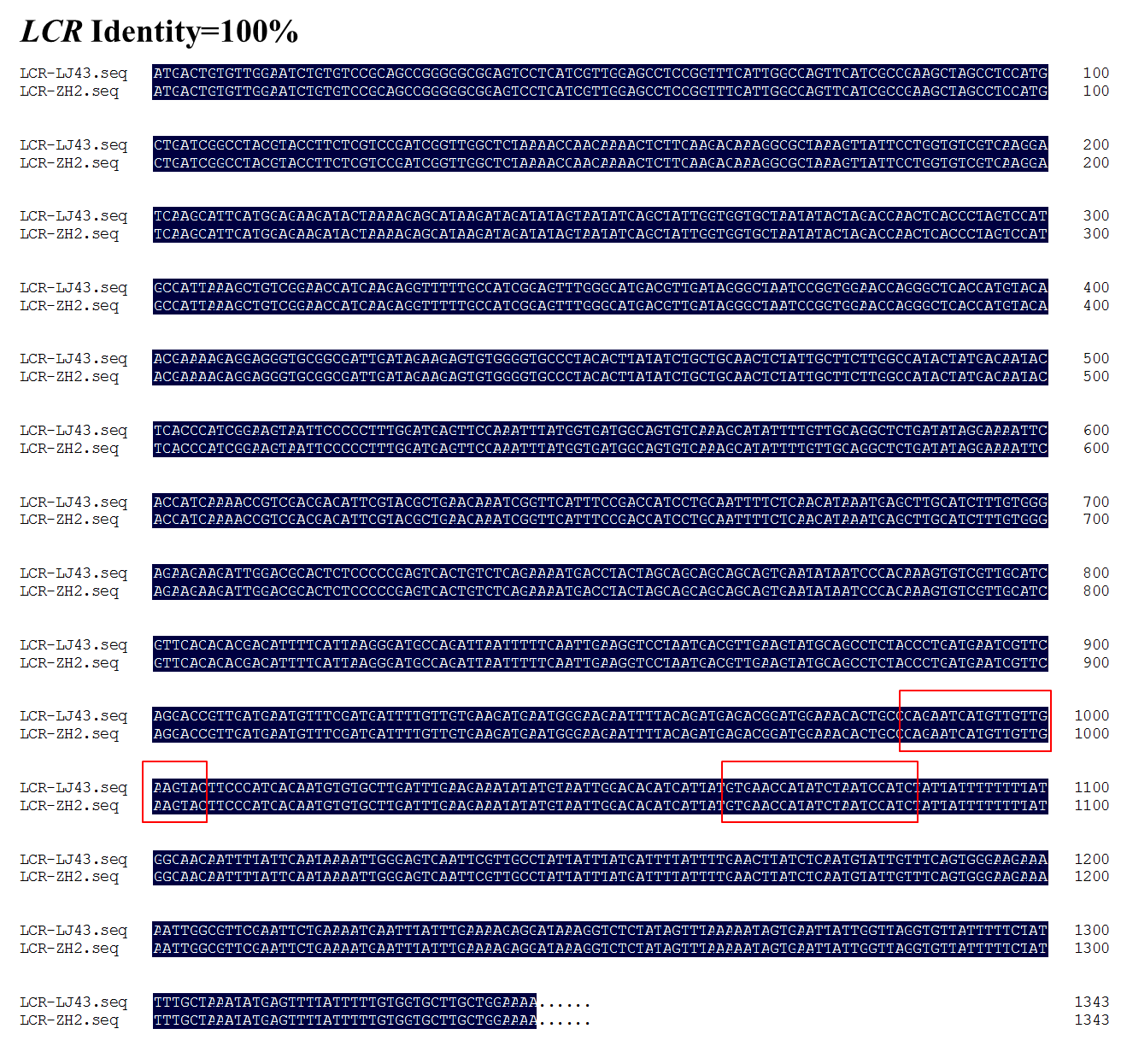
**


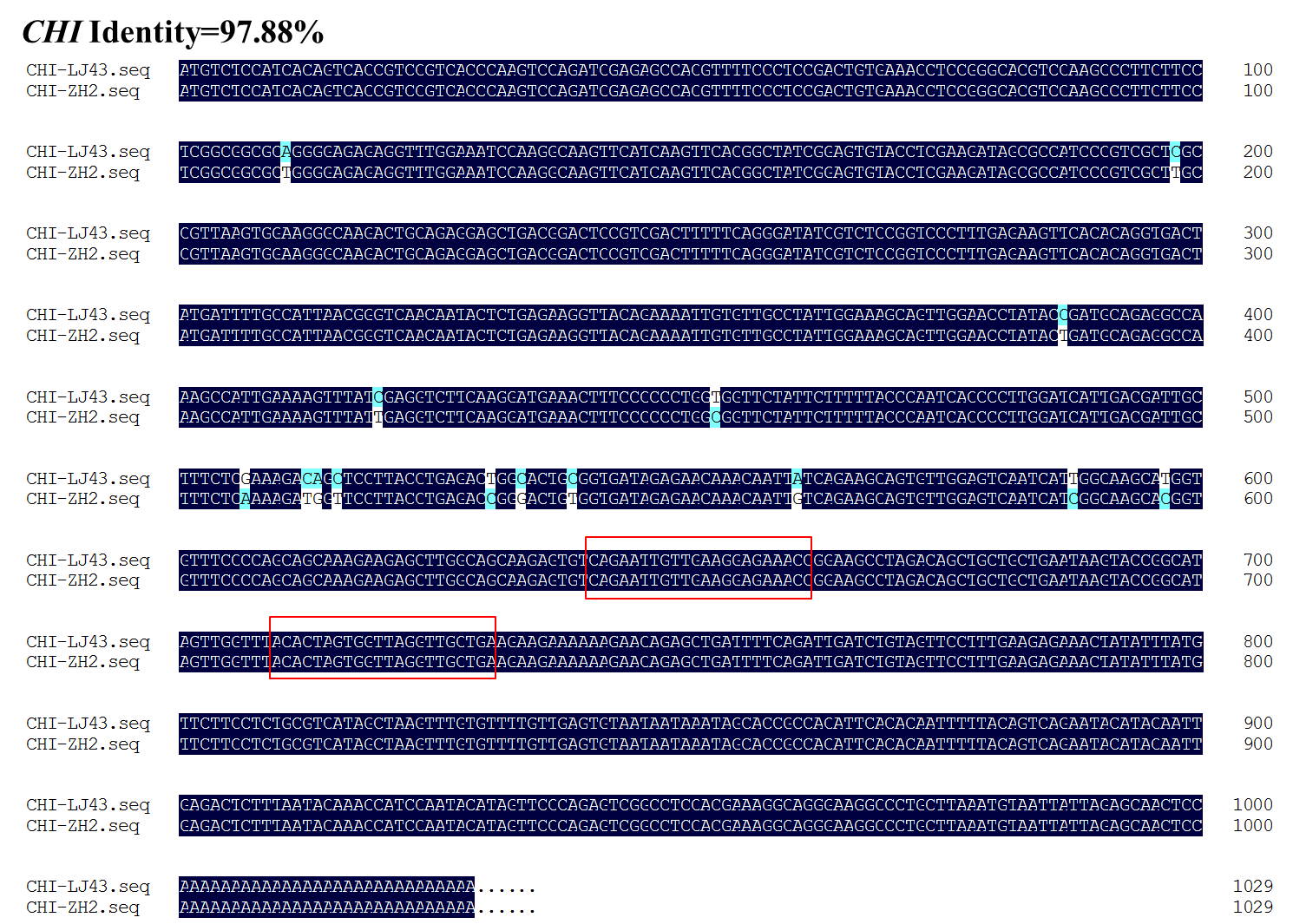


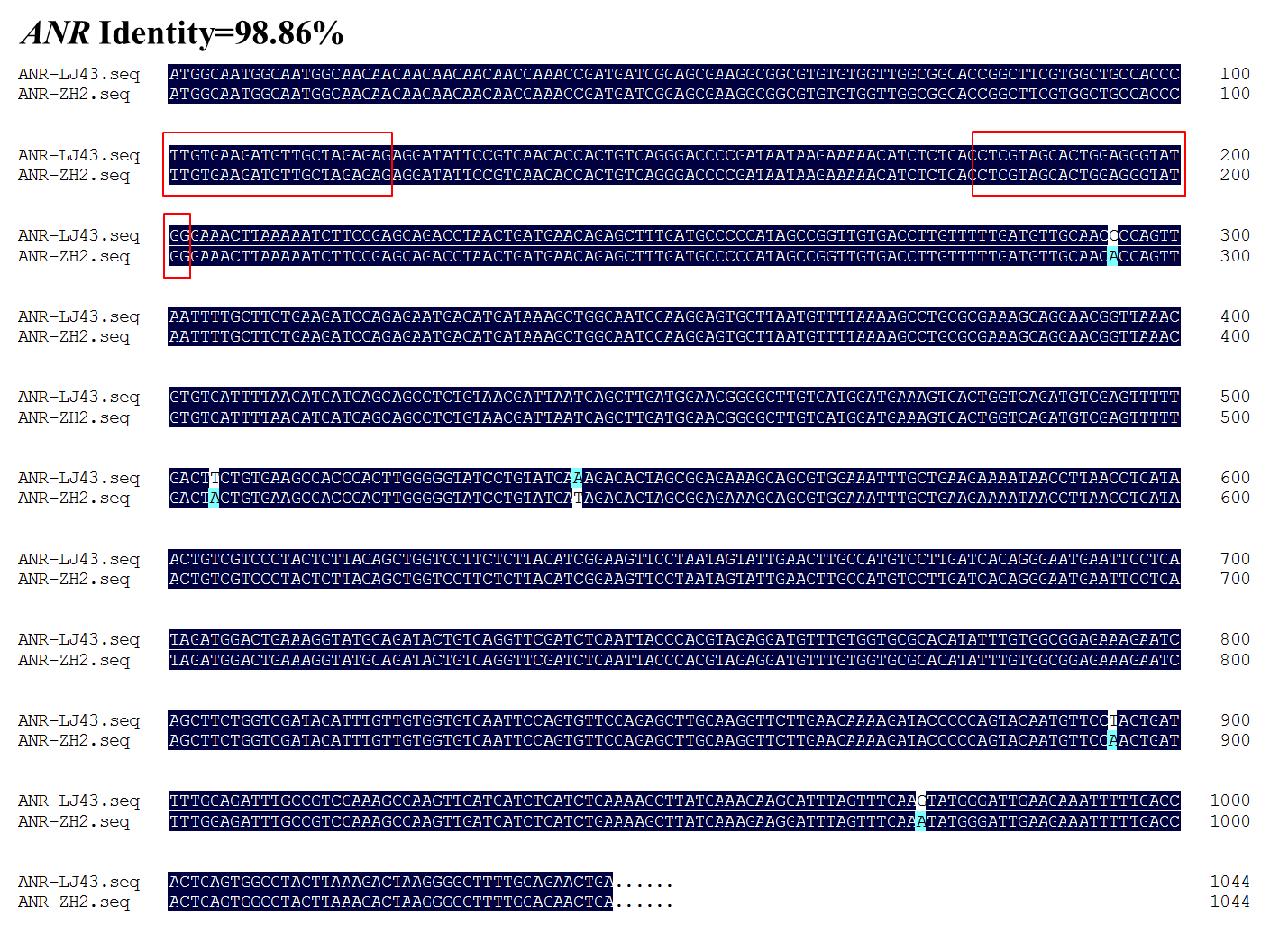


**
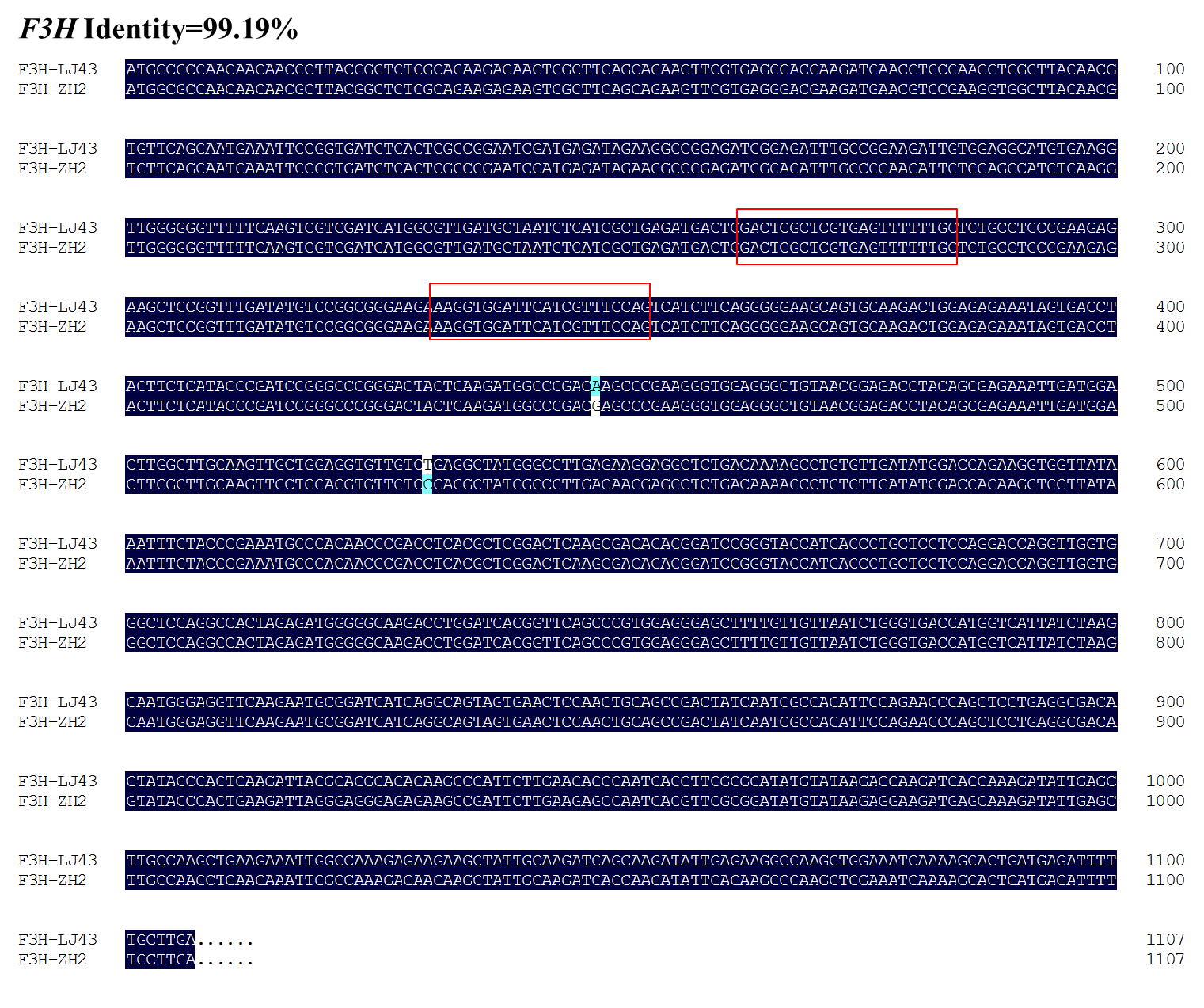
**

**
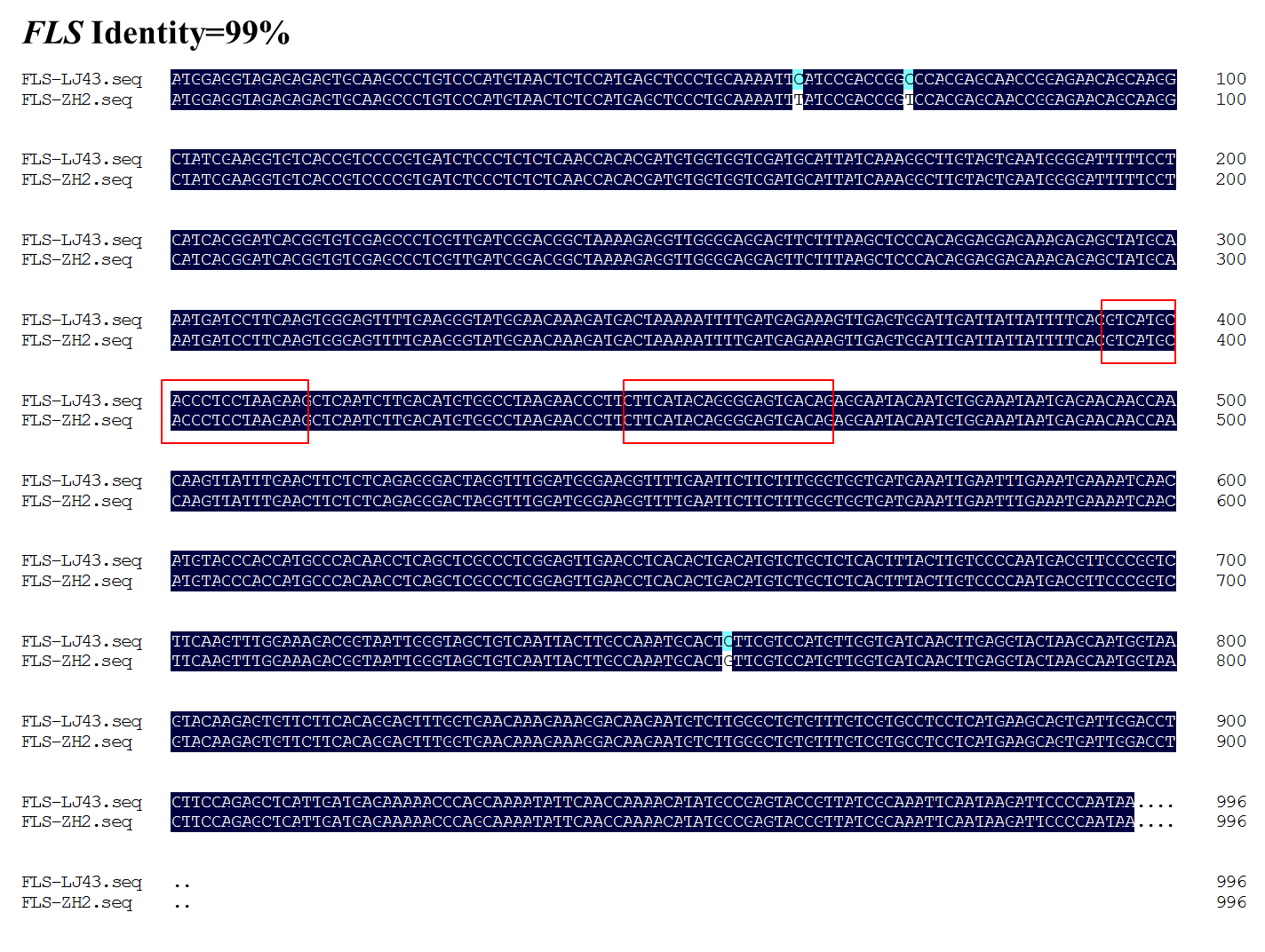
**

**
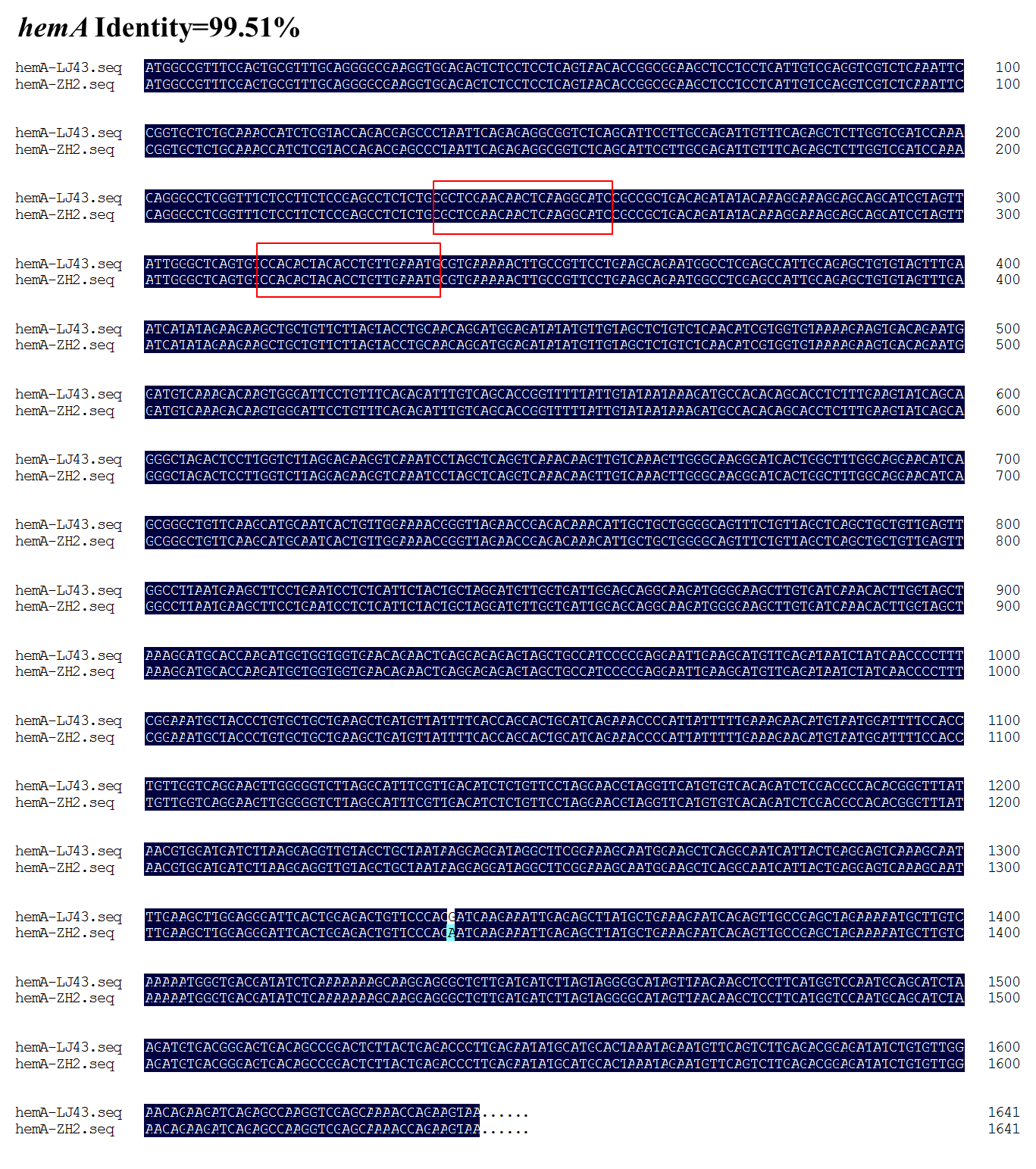
**


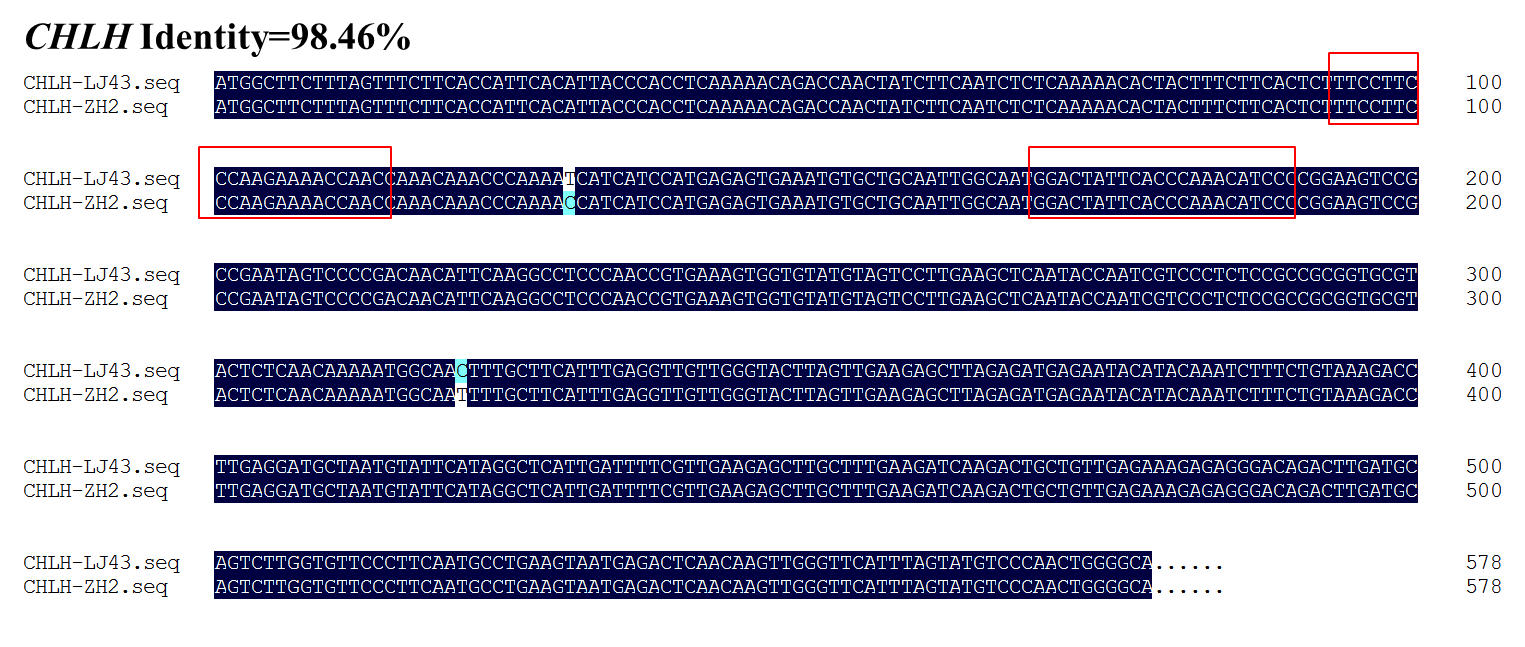


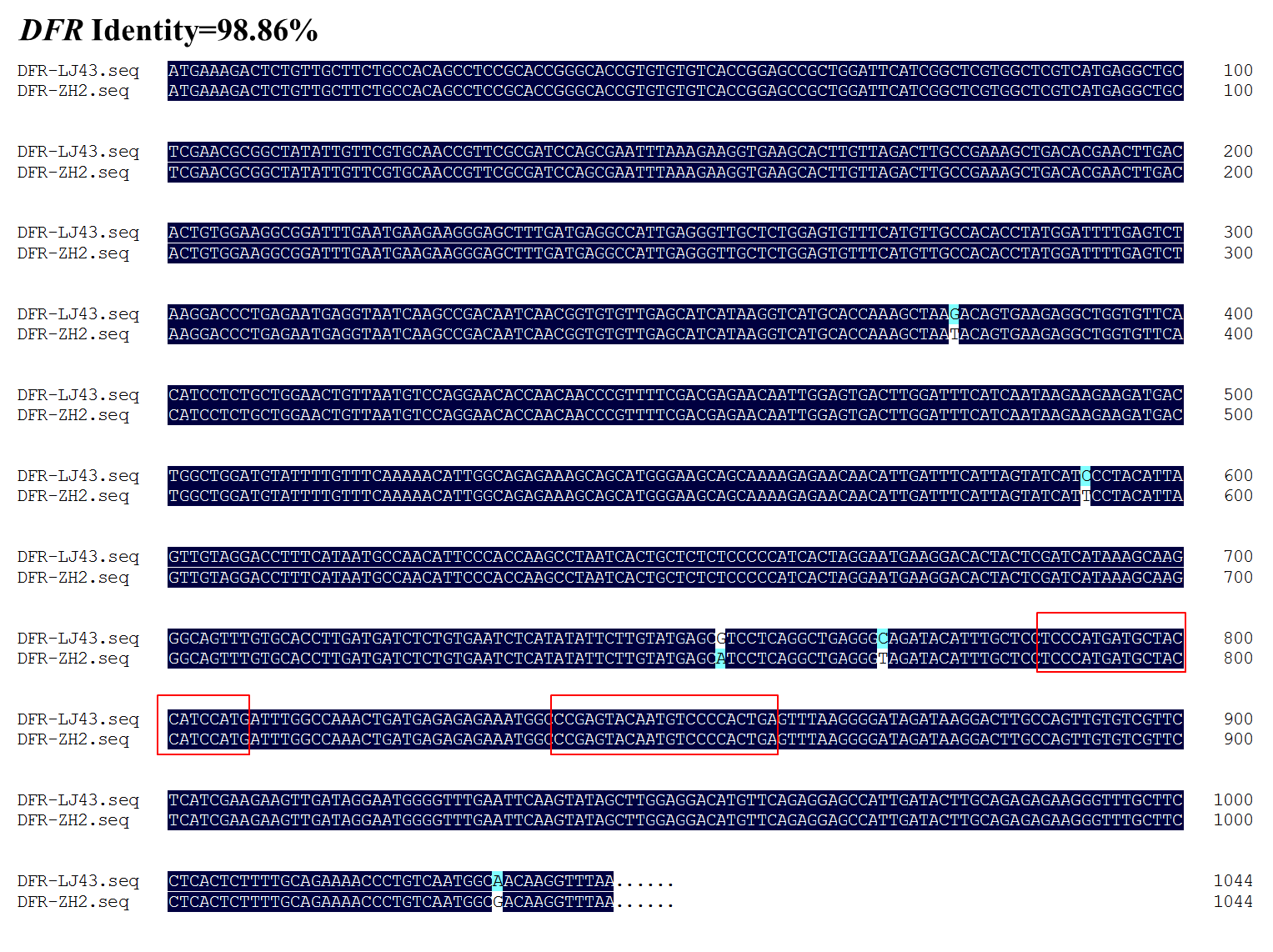


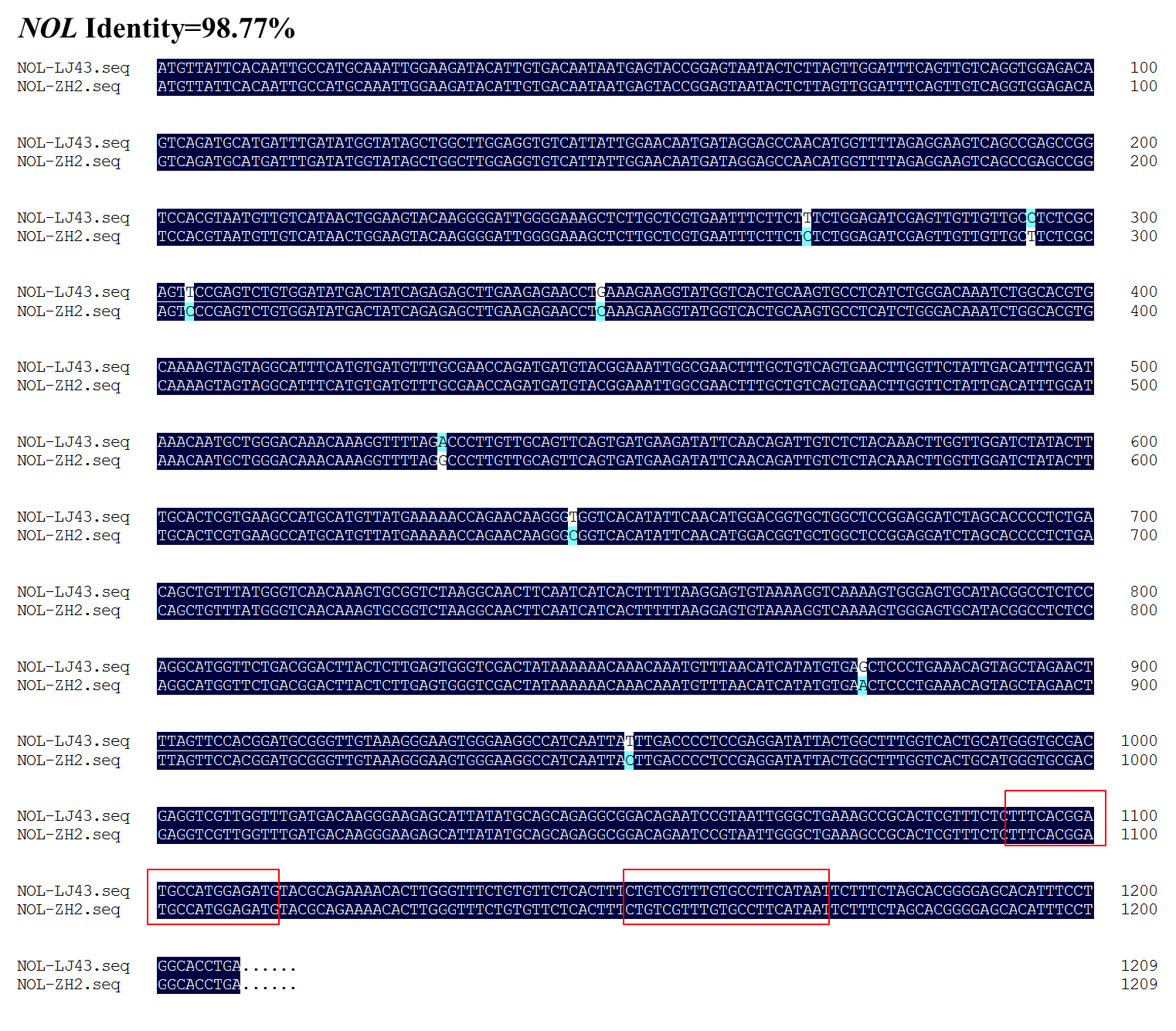


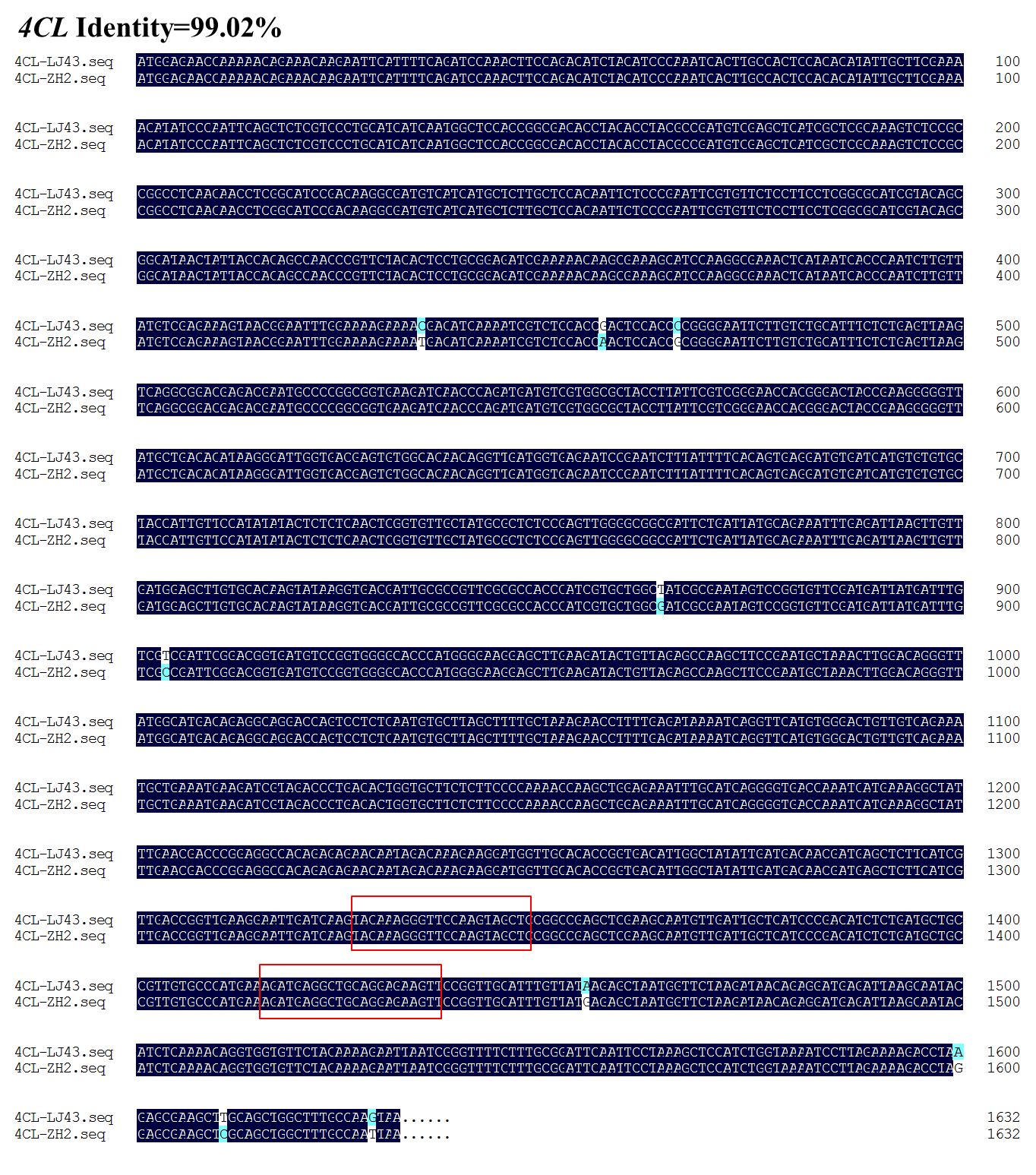

Supplement: Additional file 5: — Ten genes randomly selected from microarray data were cloned from two tea cultivars by RACE. [file 12870_2014_352_MOESM5_ESM.docx]
